# Formal procedure to facilitate the decision to withhold or withdraw life-sustaining interventions in a neonatal intensive care unit: a seven-year retrospective study

**DOI:** 10.1186/s12904-018-0329-x

**Published:** 2018-05-17

**Authors:** G. Sorin, R. Vialet, B. Tosello

**Affiliations:** 1 Department of Anesthesia and Intensive Care, Neonatal and Pediatric Intensive Care Unit, Hospital Nord, Assistance-Publique des Hôpitaux de Marseille, 13015 Marseille, France; 2 Department of Neonatology, Hospital Nord, Assistance-Publique des Hôpitaux de Marseille, 13015 Marseille, France; 3 Aix Marseille University, CNRS/EFS/ UMR 7268 ADES, Marseille, France

**Keywords:** NICU, Neonatal death, LWAT file

## Abstract

**Background:**

Neonatal deaths are often associated with the complex decision to limit or withdraw life-sustaining interventions (LSIs) rather than therapeutic impasses. Despite the existence of a law, significant disparities in clinical procedures remain. This study aimed to assess deaths occurring in a Neonatal Intensive Care Unit (NICU) and measure the impact of a traceable Limitation or Withdrawal of Active Treatment (LWAT) file on the treatment of these newborns.

**Methods:**

In this monocentric retrospective study, we reviewed all consecutive neonatal deaths occurring during two three-year periods among patients in the NICU at the North Hospital of Marseille: cohort 1 (from 2009 to 2011 without the LWAT file) and cohort 2 (from 2013 to 2015 after introduction of the LWAT file). Newborns included were: gestational age over 22 weeks, birth weight over 500 g, and admission and death in the same NICU. Deaths were categorized according to the classification described by Verhagen et al.: 1) children who died despite cardiopulmonary resuscitation (CPR) (no withholding nor withdrawing of LSIs), (2) children who died while the ventilator, without CPR (no withdrawing of LSIs, but CPR withheld), (3) children who died after LSIs were withdrawn, or (4) LSIs were withheld.

**Results:**

193 deaths were analyzed: 77 in cohort 1 and 116 in cohort 2. 50% of deaths followed the decision to limit or stop life-sustaining interventions. The mean age at death did not differ between the two cohorts (*p* = 0.525). An increase in the mortality rate after life-sustaining interventions were withdrawn was observed. The number of multidisciplinary decision meetings was statistically higher in cohort 2 (32.5% versus 55.2% *p* = 0.002), which were most often prompted due to neurological pathologies, with an increase in parental advice concerning the management of their child (*p* = 0.026). Even if the introduction of this file did not have an effect on patient age at death, it was significantly associated with a better understanding of end-of-life conditions (*p* = 0.019), including medication used to sedate and comfort the patient.

**Conclusions:**

Introduction of the LWAT file seems imperative to develop a personalized healthcare strategy for each child and situation.

**Electronic supplementary material:**

The online version of this article (10.1186/s12904-018-0329-x) contains supplementary material, which is available to authorized users.

## Background

Many debilitated newborns survive thanks to recent innovations and technical advances in neonatology. Nevertheless, serious complications, especially neurological conditions, may cause physicians to make complicated decisions to either limit or withdraw treatment instead of continuing intense curative interventions [1–4]. Neonatal deaths are rarely due to a technical impasse; instead, they are often associated with a decision to limit or withdraw active therapy (14% versus 69-93%, respectively) [5–7]. While the medical field continues to advance and push the limits of healthcare treatment possibilities, extending an individual’s lifespan often implies a compromised quality of life (QoL). In these often uncertain situations, decision-making is influenced by the consequences of QoL and possibilities of severe disability, which undoubtedly raises ethical questions [8–10].

The French law of 22 April 2005 [11], which concerns the rights of patients and at the end of life, provides a regulatory framework for professionals in neonatal medicine and enables an adapted response for the majority of situations involving end-of-life procedures concerning newborn patients. Palliative care is centered on the individual, providing multidisciplinary treatment focused on the patient’s physical, psychological, social and spiritual needs, as well as family support before and after the patient passes away. Palliative care should not cause death but rather preserve the best QoL until death. The palliative approach constitutes a legal support for caregivers. Indeed, before such laws were established, caregivers provided unstructured palliative care, which unfortunately led some caregivers to totally abandon treatment or even induce deliberate and clandestine death. In France, 73% of neonatologists surveyed via anonymous questionnaire have reported administering drugs with the intention of ending a patient’s life (the same survey found 47% in the Netherlands and less than 5% in the five other countries included in the EURONIC study) [12]. Ten years later, Garel et al. reported more communication with parents in the decision-making process and more transparency on end-of-life procedures [13]. If curative treatment is no longer efficient or causes intense patient suffering, end-of-life procedures must be considered. In neonatal medicine, palliative care approaches are imposed on neonates following an initial intervention of active resuscitation with a radiological (in particular neurological) assessment, which is detrimental to their QoL.

Despite the legal context governing caregiver procedures [11, 14], marked disparities have been observed in neonatal unit [15–18]. However, published reports of mortality rates fail to specify the details and management of each death. Therefore, the current study aimed to investigate newborn deaths in a level 3 Neonatal Intensive Care Unit (NICU) and evaluate the impact of the LWAT file (Limitation or Withdrawal of Active Treatment) on the management and treatment of these newborns.

## Methods

In this retrospective monocentric study, we reviewed all consecutive deaths occurring during two three-year periods among newborns cared for at our NICU from January 1, 2009 to December 31, 2011 (cohort 1, without LWAT file) and from January 1, 2013 to December 31, 2015 (cohort 2, after introduction of the LWAT file). This project was approved by the University of Aix-Marseille Ethics Committee (n ° 2017-01-03-011).

Approximately 2500 children are born each year in our center, which includes a 16-bed Neonatal and Pediatric ICU and a 25-bed Neonatal Unit. The center is included in the Mediterranean PACA-Corsica-Monaco Perinatal Network, which coordinates and reinforces the members in the interregional zone to better support and improve pre-conception conditions, the course of pregnancies, birth conditions, and the well-being of both the children and their parents. The NICU admits an average 400 patients a year.

The study inclusion criteria were: gestational age over 22 weeks, a birth weight over 500 g, and hospitalization and death in the same NICU. Information was collected in each chart regarding demographic characteristics, associated pathology, details of the decision-making process, end-of-life procedures based on the child’s medical file, and the LWAT file details when possible (from 2013 onward).

According to the law: “*In case the patient is unable to express his/her will, the termination of treatment can no longer depend on the patient’s choice, and it is thus necessary to seek alternative legal conditions; the only case in which it is authorized by law, is the unreasonable refusal of obstinacy mentioned in Article L.1110-5. This article cites cases in which treatments appear to be unnecessary, disproportionate or have no effect other than to artificially maintain life*”. The physician must implement a so-called collegiate reflection procedure (Decree No. 2006-120 of February, 6 2006, Decree No. 2010-107 of January 29, 2010). The main directives derived from the LEONETTI law of April 22, 2005 are:

- Ratification of ability to refuse extraordinary therapeutic measures (Art. 1),
- More rigorous enforcement of provision of information to patients (Art. 2),
- More rigorous enforcement of freedom of choice accorded to conscious patients (Art. 6),
- The introduction of the concept of “double effect”,
- The introduction of the “collegial procedure” for patients unable to express their wishes,
- The decriminalization of limitation of treatments (Art. 122-4 of the French Criminal Code) provided procedure is adhered to,
- Ratification of palliative care (extended to community-based health care establishments and more rigorously enforced in health care institutions).

The French law of April 22, 2005 on patient rights and end-of-life reinforces the rights of all patients and accords specific rights to end-of-life patients. The practitioner must support the patient until the patient’s final moments, ensure quality of life through appropriate care and measures, protect the patient’s dignity, and comfort the patient’s entourage. The practitioner does not have the right to deliberately cause death (Art. R.4127-38 of the French Public Health Code). As regards situations of lethal fetal abnormality, this law applies to the unborn baby.

Identification of unreasonable obstinacy pre-birth, the first phase of palliative care, 6 appears a vague and potential concept (anticipating unreasonable obstinacy at the neonatal stage). Unreasonable obstinacy is defined in French law as care that is futile, disproportionate or has no effect other than to artificially prolong life, equivalent to the concept of “futility” in American law. Whatever the probability of the initial diagnosis being correct, it completely takes over parental subjectivity. The newborn acquires the legal status of a person; they too can die and the law makes it possible for them to die supported, relieved, and surrounded.

The LWAT file, which serves to assist the physicians, was implemented in our center in 2012. The current third version of the LWAT file is the result of a multidisciplinary deliberation within our center (see Additional file 1). The LWAT file used is filled out in real time during the multi-disciplinary meeting by the doctor responsible for the child. Each item is discussed collegially and the result of the discussion transcribed. Parents do not attend the meeting but are notified in advance of the meeting.

When the LWAT file was introduced for the management of neonatal palliative care, we established a year-long statistical washout period to avoid confusion bias. Deaths were categorized according to the classification described by Verhagen et al. [18]: (1) children who died despite cardiopulmonary resuscitation (CPR) (no withholding nor withdrawing of LSIs), (2) children who died while the ventilator, without CPR (no withdrawing of LSIs, but CPR withheld), (3) children who died after LSIs were withdrawn, or (4) LSIs were withheld.

Statistical analyzes were performed using the SPSS 20 software. Descriptive statistics were used; expressed in numbers and percentages; the corresponding averages and standard deviations (+/− SD) or median and interquartile (IQR) were also indicated where appropriate. To compare our data, we used the Pearson chi2 test or the Fisher’s exact test (with less than five data points). In our study, a *p*-value of < 0.05 was considered to indicate statistical significance.

## Results

There were a total of 193 neonatal deaths: 77 in cohort 1 and 116 in cohort 2. The characteristics of our population are outlined in Table 1. We did not observe a significant difference between these two groups. Only the place of birth was significant between the two cohorts (*p* = 0.014), as cohort 1 included more newborns who were outborn and then transferred to our NICU.

**Table 1:** Demographic characteristics

	Cohort 1 (*n* = 77)	Cohort 2 (*n* = 116)	*p* value
Gestational age, mean (+/− SD) (weeks)	30 (5)	29 (5.1)	0.451
Weight, mean (+/− SD) (g)	1508 (1056)	1435 (994)	0.666
Sex, n (%)			
Female	36 (46.8)	50 (43.1)	0.617
Place of birth, n (%)			
Inborn	56 (72.7)	64 (55.2)	0.014
Resuscitation in delivery room, n (%)			
Yes	63 (81.8)	89 (76.7)	0.397
Apgar < 7 to 5 min, n (%)	34 (44.1)	60 (51.7)	0.853
Birth context, n (%)			
Perinatal asphyxia	8 (10.4)	12 (10.3)	0.674
Congenital anomaly	14 (18.2)	13 (11.2)	
Premature	17 (22.1)	25 (21.6)	
Weight < 1000 g	37 (48.1)	63 (54.3)	
Other	1 (1.3)	3 (2.6)	
Age at death, mean (+/− SD) (days)	19 (51)	15 (21)	0.525
Age at death, median (IQR) (days)	6 (16)	7 (16)	
Death delay time, n (%)			
Within 48 h	21 (27.3)	21 (18.1)	0.131
In the first week	22 (28.6)	42 (36.2)	0.270
Within three weeks	20 (26)	29 (25)	0.879
Within two months	7 (9.1)	17 (14.7)	0.251
Within three months	4 (5.2)	3 (2.6)	0.343
Antenatal palliative care decision, n (%)	1 (1.3)	0	0.218
Verhagen classification, n (%)			
Class 1	12 (15.6)	15 (12.9)	0.061
Class 2	35 (45.5)	34 (29.3)	
Class3	10 (13)	16 (13.2)	
Class 4	20 (26)	50 (43.1)	
Cause of death, n (%)			
Severe hypoxic-ischemic encephalopathy	9 (11.7)	13 (11.2)	0.708
Other neurological injury	16 (20.8)	35 (30.2)	
Sepsis	16 (20.8)	17 (14.7)	
Hemodynamic failure	19 (24.7)	29 (25)	
Congenital anomaly	7 (9.1)	10 (8.6)	
Respiratory failure	10 (13)	11 (9.5)	
Other	0	1 (0.9)	
Collaborative decision meeting, n (%)	25 (32.5)	64 (55.2)	0.002

Verhagen classification: class 1: children who died despite cardiopulmonary resuscitation (CPR) (no withholding nor withdrawing of LSIs), class 2: children who died while the ventilator, without CPR (no withdrawing of LSIs, but CPR withheld), class 3: children who died after LSIs were withdrawn or class 4: LSIs were withheld

### Modes and timing of deaths

**Fig. 1:**
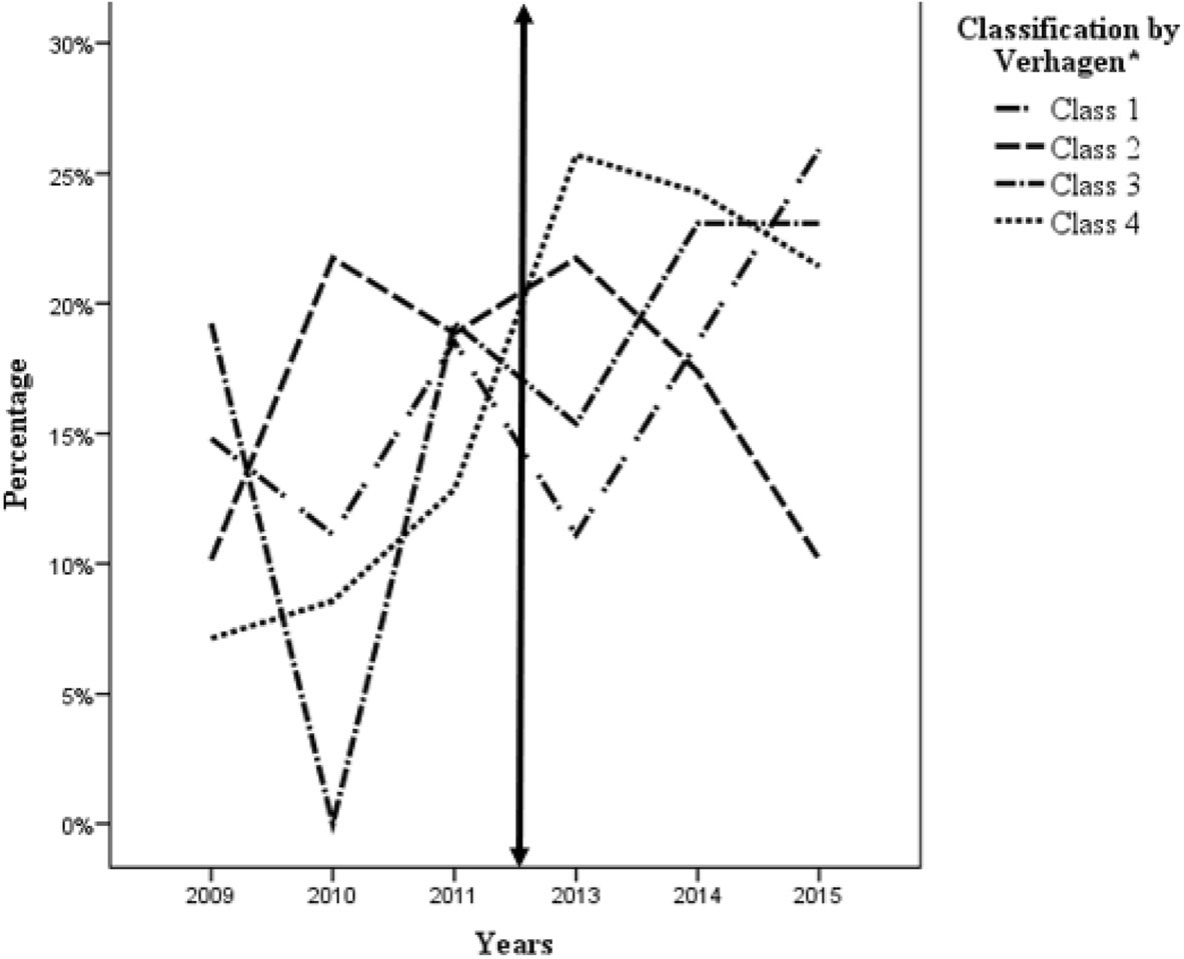
Evolution of deaths according to the Verhagen classification. Verhagen classification: Class 1: death despite cardiopulmonary resuscitation; Class 2: death despite the maintenance of treatment, ventilation but without CPR; Class 3: death after limiting treatment, and Class 4: death after stopping treatment. Two-sided arrow: introduction of the LWAT file

The mean age at death (19 days for cohort 1 versus 15 days for cohort 2) did not significantly differ (*p* = 0.525). We did not find a significant difference in the cause of death in the two groups or in their distribution according to the Verhagen classification. Figure 1 shows the evolution of the Verhagen class by year. Class 1 (maximum treatment with CPR) and class 2 (maximum treatment but no CPR) remained relatively stable over time, accounting for approximately 20 and 10% of deaths per year, respectively. After a sharp decline in 2010, class 3 (withdrawing LSIs) appeared to stabilize at 20-25% of deaths. By contrast, a clear increase in the mortality rate after withholding LSIs (class 4) was observed, from 8% of deaths in 2009 (nadir) to 25% of deaths in 2013.

### Collaborative decision meeting (Table 2)

Discussions were statistically higher in the second period (32.5% versus 55.2%, *p* = 0.002) even though the number did not differ between the two groups. In spite of the lack of statistical significance in “pathology that prompted discussions” (*p* = 0.705), we noted a higher rate of pathologies involving neurological lesions. There was no significant difference in the average time between the date of birth and the meeting, the decision made at the meeting, the parents’ opinion, or even the time between the meeting and the child’s death. By contrast, the collecting of the parents’ opinions concerning the care of their child before multidisciplinary meetings was significantly higher in cohort 2 (*p* = 0.026).

**Table 2:** Characteristics of Collaborative decision meeting

	Cohort 1 (*n* = 25)	Cohort 2 (*n* = 64)	*P* value
Number of meetings, n (%)			
One	24 (96)	58 (90.6)	0.084
Two	0	6 (9.4)	
Three	1 (4)	0	
More than three	0	0	
Pathology prompting meeting, n (%)			
Severe hypoxic-ischemic encephalopathy	5 (20)	11 (17.2)	0.705
Other neurological injury	12 (48)	36 (56.3)	
Sepsis	1 (4)	4 (6.3)	
NEC	1 (4)	0	
Congenital anomaly	3 (12)	7 (10.9)	
Acute respiratory disease	0	1 (1.6)	
Other	3 (12)	5 (7.8)	
Time delay between birth and meeting, mean (+/− SD) (days)	26 (33.5)	18 (24.4)	0.494
Parental opinion known, n (%)	4 (16)	27 (42.2)	0.026
Decision at the end of the meeting, n (%)			
Continue treatment	1 (4)	0	0.259
Limit treatment	7 (28)	20 (31.3)	
Withdraw treatment	16 (64)	44 (68.8)	
Parental opinion after meeting, n (%)			
Agreement	24 (96)	57 (89.1)	0.325
2003Time delay between meeting and death, median (IQR) (days)	1 (1.0)	1 (1)	0.839
Completed LWAT file, n (%)			
	0	52 (81.3)	< 0.001

### Effect of the LWAT file (Table 3)

The introduction of the LWAT file during the second study period (cohort 2) did not have a significant effect on age at death or life expectancy in relation to the pathology. Furthermore, introduction of the file was not more frequently associated with a specific context or pathology. However, these records were significantly associated with a better understanding of end-of-life conditions (*p* = 0.019): type and description of LSIs (CPR, limitation of ventilation parameters, surgery, dialysis, antibiotic therapy, transfusion, amine, central venous catheter, extubation; parenteral nutrition, enteral nutrition, or paraclinic examination; *p* < 0.001). If morphine use was comparable between the two periods, the use of morphine-associated hypnotics during end-of-life management increased between 2013 and 2015 (20% versus 48.1%, respectively; *p* = 0.018). The use of respiratory support (mechanical ventilation or continue positive airways pressure (CPAP) at the time of death did not differ between the periods.

**Table 3:** Impact of the LWAT file on end-of-life management

	Cohort 1 with meeting (*n* = 25)	Cohort 2 Meeting + file (*n* = 52)	*p* value
Age at death, mean (+/− SD), median (IQR) (days)	28.3 (36.4) 14 (36)	21.7 (25.6) 11 (17.5)	0.556
Death < 1 week, n (%)	8 (32)	17 (32.7)	0.952
Birth context, n (%)			
Severe hypoxic-ischemic encephalopathy	4 (16)	9 (17.3)	0.445
Congenital anomaly	5 (20)	5 (9.6)	
Premature	7 (28)	11 (21.2)	
Weight less than 1000 g	9 (36)	27 (51.9)	
Verhagen classification, n (%)			
Class 1	0	1 (1.9)	0.654
Class 2	1 (4)	3 (5.8)	
Class 3	7 (28)	9 (17.3)	
Class 4	17 (68)	39 (75)	
Parental opinion before meeting	4 (16.7)	25 (48.1)	0.009
Pathology prompting the meeting, n (%)			
Severe hypoxic-ischemic encephalopathy	5 (20)	8 (15.4)	0.742
Other neurological disease	12 (48)	30 (57.7)	
Sepsis	1 (4)	3 (5.8)	
Congenital anomaly	3 (12)	6 (11.5)	
Acute respiratory disease	1 (4)	1 (1.9)	
Other	3 (12)	4 (7.5)	
Cause of death, n (%)			
Severe hypoxic-ischemic encephalopathy	4 (16)	9 (17.3)	0.774
Other neurological injury	13 (52)	27 (51.9)	
Sepsis	1 (4)	2 (3.8)	
Hemodynamic failure	2 (8)	7 (13.5)	
Congenital anomaly	4 (16)	6 (11.5)	
Respiratory failure	1 (4)	1 (1.9)	
Lifetime according to pathology, median (IQR)			
Severe hypoxic-ischemic encephalopathy	13 (14.5)	11 (5.5)	0.337
Congenital anomaly	20 (112.5)	31 (41)	
Premature	15 (37)	11 (21)	
Weight < 1000 g	11 (54.5)	9 (17)	
Understanding of end-of -life conditions, n (%)	21 (84)	51 (98.1)	0.019
Decision at the end of the meeting, n (%)			
Continue treatment	1	0	0.318
Withhold treatment	7	14 (26.9)	
Withdraw treatment	16	38 (73.1)	
Sedation, n (%)	24 (96)	49 (94.2)	0.743
Hypnotics	5 (20)	25 (48.1)	0.018
Morphine	23 (92)	48 (92.3)	0.962
Ventilation support at time of death, n (%)	6 (24)	12 (23.1)	0.929
Mechanical ventilation	5 (20)	11 (21.2)	0.864
CPAP	1 (4)	1 (1.9)	
High flow nasal canulea	0	0	

Verhagen classification: class 1: children who died despite cardiopulmonary resuscitation (CPR) (no withholding nor withdrawing of LSIs), class 2: children who died while the ventilator, without CPR (no withdrawing of LSIs, but CPR withheld), class 3: children who died after LSIs were withdrawn or class 4: LSIs were withheld

## Discussion

In this study, we aimed to investigate all deaths occurring in our NICU and evaluate the effect that the LWAT file had on neonatal deaths. We focused exclusively on deaths in the ICU and excluded deaths reported in delivery room, where decisions are often based not only on the gestational age of the newborn, as suggested by the EPIPAGE-2 study by Perlbarg et al. [19].

On average, 50% of deaths in our study followed a decision to withdraw or withhold LSIs, according to the literature [2–4, 12, 20]. The low variability of this mortality rate over time or in relation to implicated pathology was also observed by Dupont-Thibedeau et al. [4], which could lead to the development of habits concerning end-of-life issues in the ICU instead of personalized decisions made on a case-by-case basis. While the age at death reported by Dupont-Thibedeau et al. [4] appeared to vary over time, no difference was observed between our two cohorts, without any identified causal factor. Nevertheless, the mortality rate after withholding LSIs (Verhagen class 4) in our study increased over time, a trend that has also been observed in several countries (e.g., Northern Europe and North America) [3, 4, 15, 18, 21]. However, only a slight increase has been reported in Taiwan [16] and Latin America [17], thus reflecting cultural and religious differences in the recognition of unreasonable obstinacy for example [22].

In our population, neurological abnormalities were predominantly the pathologies that prompted collaborative decisions meetings, which raises the issue of QoL. Hellmann et al. [3] and Verhagen et al. [18] have respectively attributed 41% and 19-35% of deaths to the withdrawal of active therapy after an undeniably subjective estimate of potential “poor QoL” in the future. Physicians and parents seem to accept a certain degree of uncertainty in the decision to limit or discontinue treatment. According to Ceccaldi [23], this situation can be paralyzing, preventing the physician from making a decision or, in extreme cases, forcing the physician to base the decision on science, a religious reference or personal convictions. This inherent and fundamental uncertainty concerning the future of these children presents a limited margin for decisions [3, 18, 24].

We reported a limited number of meetings involving the final decision per child, whereas this figure appears to be greater in the literature [3]. Based on these decisions, the main method applied to discontinue therapy was termination of mechanical ventilation associated with sedation [4, 25]. Almost 95% of patients received analgesia at the time of death, which is higher than the rates reported in the literature [12, 16, 19, 25]. This medication has evolved over the years, with hypnotics increasingly associated with morphine. The recent law of February 2, 2016, known as the Claeys Leonetti law [14] is in line with the 2005 law [11], although it includes new rights, such as the right to profound and continuous sedation. The law provides three exceptional circumstances for which the physician must implement this specific sedation until the patient dies to avoid suffering: (1) when the patient cannot express his/her will and the physician, after completing a collegiate procedure, discontinues life-sustaining treatment, (2) as a result of the refusal of unreasonable obstinacy by authorizing the establishment of this sedation, and (3) situations that can be transferred to the field of neonatology [26]. Even if this law supports a true culture and politics of palliative care, certain issues still remain unclear, especially concerning the pediatric population. The introduction of the LWAT file, in response to the legal requirements, is a guide and support for health professionals, enabling them to better document the decision-making processes [25, 27]. In our cohort, use of the LWAT file did not have an effect on newborn age at death, which could have fundamentally challenged the very definition of the palliative approach, i.e., to avoid causing death and preserve the best possible QoL until death. The file enables physicians and other health care professionals to refine palliative methods, which is not only limited to the question of avoiding resuscitation, but also enables contemplation on an individual basis regarding the continuation/discontinuation of certain treatments or investigations while respecting the dignity of the patient. The purpose of this file is to propose a care strategy adapted specifically to the child (and his/her family) at the end of life. Nevertheless, Cremer et al. [28] have noted certain weaknesses concerning the transmission of such medical information, which for example does not enable exchange between the professionals concerned. From the beginning of the pediatric care process, parental advice is increasingly collected [25, 27, 29–31]; parents thus act as spokespeople and protectors of the best interest of their child (to assert autonomy). Encouraging the parents to express themselves and integrating them into the healthcare process guarantees trust and strengthens their sense of parenthood.

This study has certain limitations primarily due to its retrospective and monocentric characteristics.

## Conclusions

Decisions concerning the withdrawal or withholding of LSIs is often influenced by considerations of quality of life and disability consequences. The use of a file, in addition to guiding health professionals in their decision-making process, seems recommended to develop a personalized care plan for each child and every situation, without standardizing this delicate process at the end of life.

## Additional file

Additional file 1: LWAT file used. (DOCX 52 kb)

## Abbreviations

CPAP: Continous Positive Airways Pressure
CPR: Cardiopulmonary Resuscitation
ICU: Intensive Care Unit
LSI: Life-sustainig interventions
LWAT: Limitation and withdrawal of active treatment
NEC: Necrotizing enterocolitis
NICU: Neonatal Intensive Care Unit
QOL: Quality of life
