# Supplementary material for: Formal procedure to facilitate the decision to withhold or withdraw life-sustaining interventions in a neonatal intensive care unit: a seven-year retrospective study
Source: BMC Palliat Care. 2018 May 17;17:76. doi: 10.1186/s12904-018-0329-x (PMC5956735; doi:10.1186/s12904-018-0329-x)
Supplement: Supplementary file 1 — LWAT file used. (DOCX 52 kb) [file 12904_2018_329_MOESM1_ESM.docx]

Limitation and withdrawal of active treatment (LWAT) file

Patient’s name:

Meeting n°:

Summary of the previous meeting:

Purpose of the meeting of the day:

**Decision:**

Yes / No

**Réflexion:**

Yes/ No

1. **Collaborative decision meeting**

**Date :**

**Time :**

**Present speakers :**

| **Function** | **Identification** |
| --- | --- |
| **Doctor refer to patient** | Name : |
| **Resident refer to patient** | Name: |
| **Nurse refer to patient** | Name : |
| **Caregiver refer to patient** | Name : |
| **Other doctors in the department** | Name : |
|  | Name : |
|  | Name : |
|  | Name : |
| **Other external doctors** | Name : |
|  | Name : |
|  | Name : |
| **Other residents** | Name: |
|  | Name: |
|  | Name : |
| **Psychologist** | Name : |
| **Other paramedics** | Name: |

1. **Clinical context**

***Summary of the clinical situation motivating the meeting:***

1. **Parental opinion**

|  | **Mère** | **Père** |
| --- | --- | --- |
| **Unknown** | yes  no | yes  no |
| **Continue treatment** | yes  no | yes  no |
| **Limit or withdraw treatment** | yes  no | yes  no |
| **No opinion** | yes  no | yes  no |
| **Decision given to doctors** | yes  no | yes  no |
| **Prior knowledge of the meeting** | yes  no | yes  no |
| **Ambiguous parents wishes** | yes  no | yes  no |

1. **Arguments for limiting treatment**

| **No longer possible curative strategy** | yes  no |  |
| --- | --- | --- |
| **The response time is sufficient to judge the inefficiency of the current strategy** | yes  no |  |
| **Expected and normal evolution of an incurable condition** | yes  no |  |
| **The prognosis is pejorative in a short time** | yes  no |  |
| **Therapeutic intervention only prolongs the survival of the patient** | yes  no |  |
| **The patient will not benefit from the therapeutic intervention** | yes  no |  |
| **The quality of future relational life will be very limited** | yes  no |  |
| **The risk of patient addiction substitutions techniques of vital functions is major** | yes  no |  |
| **L’engagement thérapeutique maximal n’est pas désiré par les parents, le considérant comme de l’obstination déraisonnable** | yes  no |  |

1. **Décision after collaborative decision meeting**

- Maximum therapeutic commitment
- Maximum therapeutic commitment due to lack of data: waiting reanimation
- Treatment limitation and palliative approach
- Stop treatment and palliative approach

Doctor signature :

1. **Application of LTAW**

- **Limitation of active treatment :**

|  | **No or stop** | | **Yes** | **Inappropriate** | | **Not discussed** |
| --- | --- | --- | --- | --- | --- | --- |
| Resuscitation cardiac arrest |  | |  |  | |  |
|  |  | |  |  | |  |
| **Ventilation :** |  | |  |  | |  |
| Intubation (ou réintubation) if respiratory distress |  | |  |  | |  |
| Non invasive ventilation (CPAP…) |  | |  |  | |  |
| Control of ventilator parameter :  (Increase *FiO2…)* |  | |  |  | |  |
| Commentaires |  | | | | | |
| Begin cathecholamine |  |  | | |  |  |
| Possible increase of cathecholamine |  |  | | |  |  |

|  | **No or stop** | **Inappropriate** | **Possible** | **Not discussed** |
| --- | --- | --- | --- | --- |
| **Transfusion** |  |  |  |  |
| **Vascular access** |  |  |  |  |
| **Antibiotics** |  |  |  |  |
| **Dialysis** |  |  |  |  |
| **Surgery** |  |  |  |  |

- **Withdraw of active treatments :**

|  | **Yes** | **No** | **Not discussed** | **Inappropriate** |
| --- | --- | --- | --- | --- |
| **Stop cathecholamine** |  |  |  |  |
| **Extubation** |  |  |  |  |
| **Stop CPAP** |  |  |  |  |
| **Stop antibiotics** |  |  |  |  |
| **Stop enteral feeding** |  |  |  |  |
| **Stop parenteral feeding**  **(out of hydratation)** |  |  |  |  |
| **Stop complementary exam** |  |  |  |  |

- **Palliative care :**

|  | **Yes** | **No** | **Not discussed** | **Inappropriate** |
| --- | --- | --- | --- | --- |
| **Hydratation** |  |  |  |  |
| **Sédation : benzodiazépines…** |  |  |  |  |
| **Analgesics** |  |  |  |  |
| **Nursing (aspiration,…)** |  |  |  |  |
| **Pain assessment** |  |  |  |  |
| **Breastfeeding** |  |  |  |  |
| **Personalized support (member of religion…)** |  |  |  |  |

1. **Meeting with parents afeter decision**

**Parents :**

Mother

Father

Both

Contacted by phone

Unreachable

**Parental wishes:**

Agreement with decision

Disagreement with decision

Other wishes:

**Members of the team present during the consultation with the parents:**

Doctor refer to patient: Dr

Resident refer to patient:

Other doctors: Dr

Nurse :

Other (function ?) :

1. **Evolution after decision**
2. **Consultation after death**

**Yes No**

| **Acceptance of the appointment** |  |  |
| --- | --- | --- |
| **Mother presence** |  |  |
| **Both parents present** |  |  |
| **Psychologist present** |  |  |
| **Consulting doctor :** |  |  |

**Summary of the meeting :**
